# Self-efficacy and self-management strategies in acute intermittent porphyria

**DOI:** 10.1186/s12913-019-4285-9

**Published:** 2019-07-03

**Authors:** Marte H. Hammersland, Aasne K. Aarsand, Sverre Sandberg, Janice Andersen

**Affiliations:** 1 Norwegian Porphyria Centre (NAPOS), Department of Medical Biochemistry and Pharmacology, Haukeland University Hospital, Post Office Box 1400, N-5021 Bergen, Norway; 2 The Norwegian Quality Improvement of Laboratory Examinations (NOKLUS), Haraldsplass Deaconess Hospital, Bergen, Norway

**Keywords:** Acute intermittent porphyria, Predictive genetic testing, Genetic counseling, Satisfaction with genetic counseling scale, Self-efficacy, General self-efficacy scale, Self-management strategies

## Abstract

**Background:**

Acute intermittent porphyria (AIP) is an inherited metabolic disease with low clinical penetrance caused by mutations in the hydroxymethylbilane (*HMBS*) gene. Although most patients experience little or no symptoms, serious attacks may include excruciating pain, severe electrolyte disturbances, paresis, and respiratory failure. Several drugs and lifestyle factors are potential attack inducers and avoiding known triggers is important to avoid symptomatic disease in both patients and genetically predisposed carriers. Our aim in this study was to describe self-efficacy and self-management strategies in self-reported symptomatic and asymptomatic *HMBS* mutation carriers, and to elucidate motives for predictive genetic testing.

**Methods:**

This is a cross-sectional retrospective survey with postal questionnaires. We received responses from 140 *HMBS* carriers for the general self-efficacy scale (GSES), study-specific questions about symptoms, self-management strategies and motives for genetic testing and satisfaction with the genetic counseling scale (SCS).

**Results:**

The results indicated high levels of self-efficacy in these Norwegian *HMBS* mutation carriers. Both self-reported symptomatic and asymptomatic cases recorded changes in behavior after diagnosis, such as avoiding possible triggering drugs and aspiring recommended eating habits. They were in general satisfied with the genetic counseling they had received. The possibility to prevent disease and learn about the risk of their children was their most important motives to undergo genetic testing.

**Conclusions:**

This study indicates that continuing to provide information, counseling and education is beneficial in AIP, and that *HMBS* mutation carriers, both those self-assessed as asymptomatic and as symptomatic, are using their knowledge to avoid triggering factors.

**Electronic supplementary material:**

The online version of this article (10.1186/s12913-019-4285-9) contains supplementary material, which is available to authorized users.

## Background

Porphyrias are a group of rare, inherited metabolic disorders. Each is caused by reduced, or—in one disease—increased activity in one of the enzymes in the heme biosynthetic pathway and leads to symptoms in the form of acute neurovisceral attacks, skin lesions, or both [1]. Acute intermittent porphyria (AIP) is the most common of the acute porphyrias in most countries, with an estimated prevalence of 5.9 per million inhabitants in Europe [2]. The disease is characterized by acute attacks in the form of severe abdominal pain, in combination with pain in the back and thighs, polyneuropathy, nausea, vomiting and constipation [1, 3]. Tachycardia, hypertension, electrolyte disturbances and neurological and mental complications are also frequent. Attacks of AIP have by some patients been described as excruciatingly painful [4]. Though most patients experience only one or a few acute attacks during their lifetime, more severely affected patients report a reduced quality of life. They can experience major life event consequences such as failure to secure or loss of employment, impact on family size, increased anxiety, and depression [4–6]. AIP is an autosomal dominant disease and is caused by mutations of the *HMBS* gene. Prevalence of mutations in the *HMBS* gene is probably underestimated in the healthy population [7, 8]. Clinical penetrance has been estimated to be about 10% [9], even lower in a recent population study [10]. Studies indicate that drug use including alcohol and hormonal changes are the most frequent inducers of acute attacks [11], with additional triggers being smoking, infections, physical and psychological stress, hunger and crash dieting [12, 13]. Avoidance of these triggers is recommended both to prevent *HMBS* mutation carriers not yet having symptoms from manifesting the disease, and to reduce the frequency and severity of attacks in patients who have already had symptoms of AIP. Among behavioral measures, avoiding the use of porphyrinogenic drugs is considered the single most important effort. In addition, a balanced diet with no prolonged fasting or crash dieting is generally recommended [14]. Smoking is also advised against, as smokers have been described to have more frequent acute attacks than non-smokers [12].

The Norwegian Porphyria Centre (NAPOS) offers genetic counseling of both symptomatic patients with AIP and healthy at-risk relatives, and genetic counseling is mandatory prior to predictive genetic testing. Genetic counseling usually comprises providing information about the disease with regard to genetic and biochemical mechanisms, symptoms, treatment and self-management recommendations. Additionally, if in the setting of predictive testing, the consequence of a decision to test, or not to test, is discussed. From a clinical point of view, one of the main benefits of predictive genetic testing for AIP is the possibility of choosing a lifestyle that reduces the risk of manifest disease and allows for awareness of potential long-term complications. Several drugs and behavioral factors are potential attack inducers and prevention of the disease by avoiding known triggers is by many porphyria experts a reason to recommend genetic testing in healthy at risk relatives. However, the points of view of patients with AIP on the effect of genetic testing have been investigated only poorly. Studies evaluating health behavior after genetic testing for other diseases indicate that genetic risk assessment is unlikely to lead to changes [15]. A qualitative study investigating the experiences of young adults with AIP diagnosed as minors found that early diagnosis was perceived as advantageous, but finding motivation for changes in behavior was difficult [16].

Perceived self-efficacy is considered an important factor used to explain differences in health behavior. Self-efficacy refers to a person’s degree of optimistic and self-confident view of own abilities to deal with certain life stressors [17]. Individuals with strong self-efficacy tend to make healthier lifestyle choices [18, 19]. Genetic counseling has the potential to provide AIP patients and their families with information about self-management strategies that might help reduce the risk of developing manifest disease. However, there is lack of knowledge on whether receiving an AIP diagnosis and counseling have an impact on behavior and whether this is associated with self-efficacy in patients with AIP. This information is important to improve the quality of genetic counseling and to learn how to best provide appropriate follow-up and care for persons with AIP.

### Aims

The aim of the present study was to describe self-efficacy in self-reported symptomatic and asymptomatic *HMBS* carriers and to determine whether they implemented changes in behavior after receiving the diagnosis. Furthermore, we wanted to elucidate motives for predictive genetic testing for AIP and to investigate whether those who had received genetic counseling were satisfied.

## Methods

### Design

This study was approved by the Norwegian Regional Ethics Committee (2010/1140). This was a cross-sectional retrospective questionnaire study consisting of standardized and validated patient reported outcomes measuring self-efficacy and satisfaction with genetic counseling. In addition, 14 study-specific questions were developed to 1) assess changes in behavior following the diagnosis of AIP and 2) motives for genetic testing. The questionnaire was piloted on porphyria-educated health care workers at NAPOS.

### Participants and recruitment

**Fig. 1:**
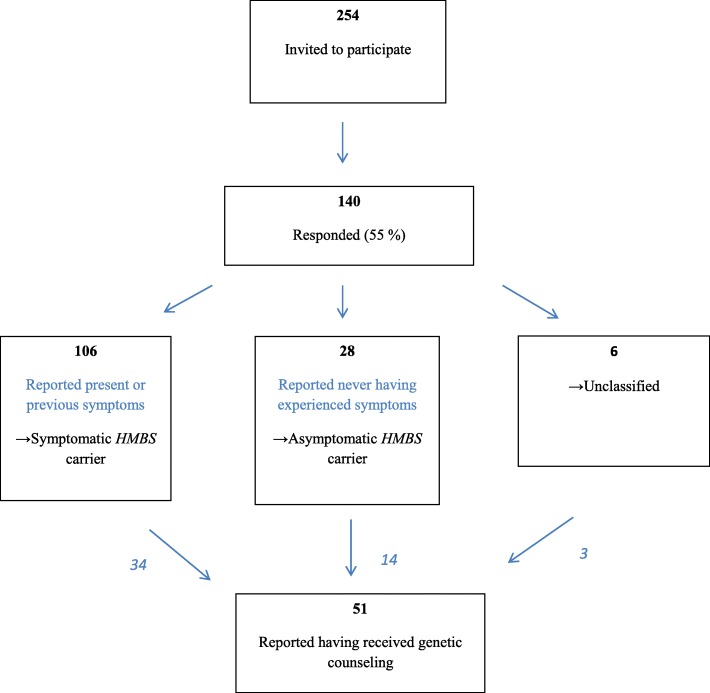
Flow chart of respondent inclusion

In May 2010, a postal questionnaire was mailed to all persons aged > 18 years registered with an AIP diagnosis at NAPOS (*n* = 254). The diagnosis of AIP was based on standard biochemical criteria [20] and/or sequencing of the *HMBS* gene as appropriate and included both those diagnosed in a symptomatic setting (investigations initiated by the treating physicians due to symptoms) and predictively tested healthy at risk relatives (investigations initiated to examine carrier status). After two follow-up reminders (June and October 2010), 140 had returned the questionnaire, giving a response rate of 55% (Fig. 1).

### Measures

#### Demographic variables and disease status

Demographic variables included: gender, age, work status, educational level, having children, and cohabitation status. Disease-related variables included self-reported symptomatic disease and whether participants had received genetic counseling. The categorization into symptomatic and asymptomatic *HMBS* mutation carriers was based on self-assessment, and reflects the participants’ subjective experiences of AIP and not an objective measure of disease status. Participants were labelled self-reported symptomatic *HMBS* mutation carrier if responding “Had previously” or “Have today/the present week” to the question “Have you ever experienced any porphyria-related complaints/symptoms?”. Participants who answered “Never had” were labelled asymptomatic *HMBS* carrier.

#### Self-efficacy and self-management strategies

The 10-item *General Perceived Self-efficacy Scale* (GSES) [17] was used to measure general self-efficacy. Self-efficacy can be defined as “beliefs in ones capabilities to organize and execute the course of action required to produce given attainments” [21], referring to a person’s perceived belief in their own ability to cope with challenges and exert control over environmental events. To determine the level of self-efficacy, all 10 items in the GSES are summed, creating a score range of 10–40, with higher scores indicating higher levels of optimistic self-belief. The scale has been found to be internally consistent and reliable across nations and languages [17]. Cronbach’s alpha value for reliability was 0.88 for the GSES in this study. To investigate to which extent the *HMBS* mutation carriers changed their awareness of triggering factors after being diagnosed with AIP, they responded to seven statements specifically constructed for our study. An example of such a statement is “I am more interested in reducing my alcohol consumption now compared with before”, where response options ranged from 1 = Not at all to 5 = Exactly true (6 = Not applicable) (Additional file 1).

#### Satisfaction with genetic counseling and motives for genetic testing

Only participants reporting to have attended genetic counseling (*n* = 51) were included in this part of the study. Participants completed the *Satisfaction with Genetic Counseling Scale* (SCS), which consists of nine items measuring three aspects of satisfaction with genetic counseling: instrumental, affective, and procedural. Instrumental satisfaction refers to how satisfied the patient is with the counselor’s professional skills and ability to explain their medical condition. Affective satisfaction reflects the psychological feedback and emotional support given by the counselor. Procedural satisfaction concerns practical matters, for example, the waiting time for the appointment. Each item is scored on a 4-point scale, creating a subscale range of 3–12, with higher scores indicating higher satisfaction. The scale has been found to hold satisfactory validity and reliability [22, 23]. Cronbach’s alpha was satisfactory for all three domains in this study: instrumental (0.86), affective (0.83), and procedural (0.87). To investigate motives for predictive genetic testing, participants were asked to respond to seven study-specific statements, such as “I wanted to clarify my own situation to facilitate prevention of the disease”, where response options ranged from 1 = Little importance to 5 = Crucial importance (6 = Not applicable) (Additional file 1).

### Statistical analyses

Statistical analyses were performed using IBM SPSS Statistics (version 22; IBM Corp., Armonk, NY, USA). Frequency (%), median, and range were calculated to describe sociodemographic variables and responses for each measurement instrument. Differences in sociodemographic variables between self-assessed symptomatic and asymptomatic participants and between those reporting having received and not received genetic counseling were investigated using χ2 test for independence for categorical variables, and one-way analyses of variance (ANOVA) for continuous variables. To describe the scores in the GSES and SCS results, the median and interquartile range (IQR; i.e., 25th and 75th percentiles), were used. Cronbach’s alpha was calculated to assess the internal consistency of the scales. The Mann–Whitney nonparametric *U* test was used to assess differences between the different groups (men vs. women; symptomatic *HMBS* vs. asymptomatic *HMBS* mutation carriers).

## Results

### Sociodemographic and disease characteristics

Of 140 respondents, 28 reported never having experienced symptoms of AIP and were labelled as self-reported asymptomatic *HMBS* carrier. Sixty-eight participants reported experiencing symptoms previously and 38 reported symptoms the current week, resulting in 106 being included in the self-reported symptomatic *HMBS* carrier group. Six respondents did not answer this question and were thus defined as “unclassified” (Fig. 1). More women than men participated in the study and the majority cohabitated and had children. Significant differences were found between the asymptomatic and symptomatic groups with regard to gender, age, and occupational status (Table 1). There were more women, higher age and more pensioners and disabled people in the symptomatic group. Within the symptomatic group, participants who had received genetic counseling reported both a higher level of education and more often being employed, compared to those who had not received genetic counseling. No such differences were observed in the asymptomatic group (Table 1). Of 114 non-responders, 63 were males and 51 women. Age ranged from 19 to 69 years, with 31 of the non-responders being 29 years or younger and 14 being 49 years or older. Based on previously registered information, 70 had experienced symptoms of AIP, 40 had never experienced any symptoms and for four no information on clinical status was available.

**Table 1:** Sociodemographic data and number of participants in the different study groups

Participant characteristics	All (*n* = 140)	*P* ^a^	Symptomatic			Asymptomatic			Unclassified (*n* = 6)
			Received GC (*n* = 34)	No GC (*n* = 72)	*p*	Received GC (*n* = 14)	No GC (*n* = 14)	*p*	
Women (%)	58	0.02	68	64	0.70	21	43	0.23	50
Age, mean (range) in years	52 (18–89)	0.03	51 (20–73)	56 (18–89)	0.10	42 (28–58)	47 (23–82)	0.03	52 (22–66)
Children (%)	79	0.47	79	82	0.76	79	71	0.66	67
Cohabiting status (%)^b^		0.36			0.16			0.80	
Living alone	21		17	25		14	14		33
Cohabitant	77		79	75		86	79		67
Highest level of education (%)		0.55			< 0.01			0.20	
Secondary school			3	33		7	29		33
High school			56	39		36	43		33
College/university			41	28		57	29		33
Occupational status (%)		0.04			0.02			0.39	
Employed^c^	62		79	47		93	79		33
Pensioner	19		3	31		0	7		33
Disabled	16		18	18		7	14		0
Other	3		0	4		0	0		33

^a^ Difference between the self-assessed symptomatic (*n* = 106) and asymptomatic HMBS mutation carriers (*n* = 28) with categorical variables analysed by χ2 test for independence, and for age by one-way analysis of variance (ANOVA)

^b^ Data not reported by two persons, one in the symptomatic and one in the asymptomatic group

^c^ Includes homeworkers and students

Differences were assessed between the symptomatic and asymptomatic group and between the subgroups of those who had reported receiving genetic counseling (GC) or not

### Self-efficacy and self-management strategies

**Fig. 2:**
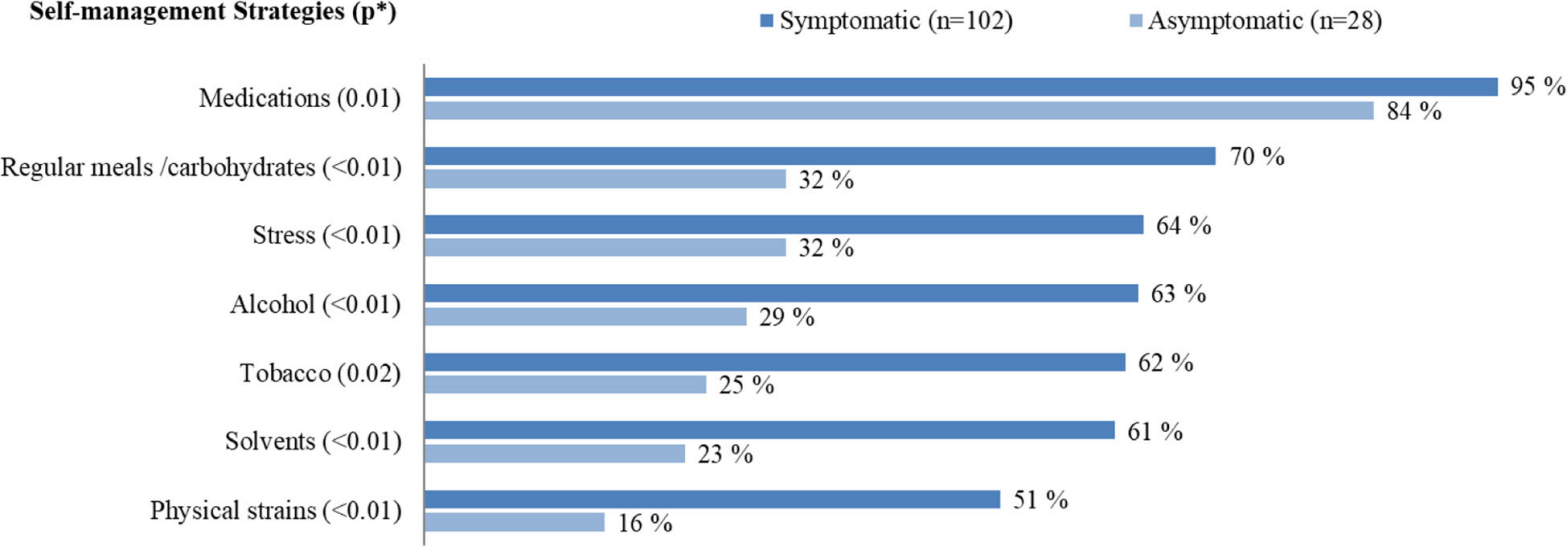
Frequency of symptomatic and asymptomatic *HMBS* mutation carriers who answered “somewhat agree” or “totally agree” to statements about changes in their awareness and concern regarding different triggers for AIP. * *p*-values were calculated with Mann-Whitney nonparametric U test. Results are presented as valid percent

The median total score on the GSES was 31 (Table 2), and no significant differences were observed between men and women or between the self-reported asymptomatic and symptomatic groups. In all groups, the median self-efficacy score was 30 or above, which is considered a high belief in ability to cope and make good choices [24]. Most respondents reported that they were more cautious with potential AIP triggers at the time of the survey compared with before receiving their diagnosis (Fig. 2). This was especially evident in regard to checking medications. More than half of the respondents also reported greater motivation for eating regular meals, avoiding stressful situations, reducing consumption of alcohol and tobacco and avoiding contact with chemical solvents. Reducing physical strains was the least common behavioral change (asymptomatic 16%, symptomatic 51%). Compared with respondents in the asymptomatic group, there was a significantly higher frequency of reported changes in behavior in the symptomatic group for all seven statements (Fig. 2). The only significant difference in lifestyle self-management between men and women was in eating habits; women were more conscious of eating regular carbohydrate-rich meals than men (*p* < 0.05, Mann-Whitney U test).

**Table 2:** General self-efficacy scale (GSES); total and subgroup scores and differences among subgroup scores

Score range 10–40			
	n	Median (IQR)	*p* ^a^
GSES total	136	31.0 (29.0, 35.0)	
Women	79	31.0 (28.0, 35.0)	0.34
Men	57	32.0 (29.0, 35.5)	
Asymptomatic *HMBS* carriers^b^	28	30.0 (29.3, 34.0)	0.86
Symptomatic *HMBS* carriers^b^	102	32.0 (29.0, 35.0)	
Unclassified	6		

*IQR* interquartile range (i.e., 25th, 75th percentiles)

^a^ By Mann–Whitney nonparametric *U* test

^b^ Categorization into symptomatic and asymptomatic *HMBS* mutation carriers was based on self-assessment by the study participants

### Genetic counseling and motives for genetic testing

**Fig. 3:**
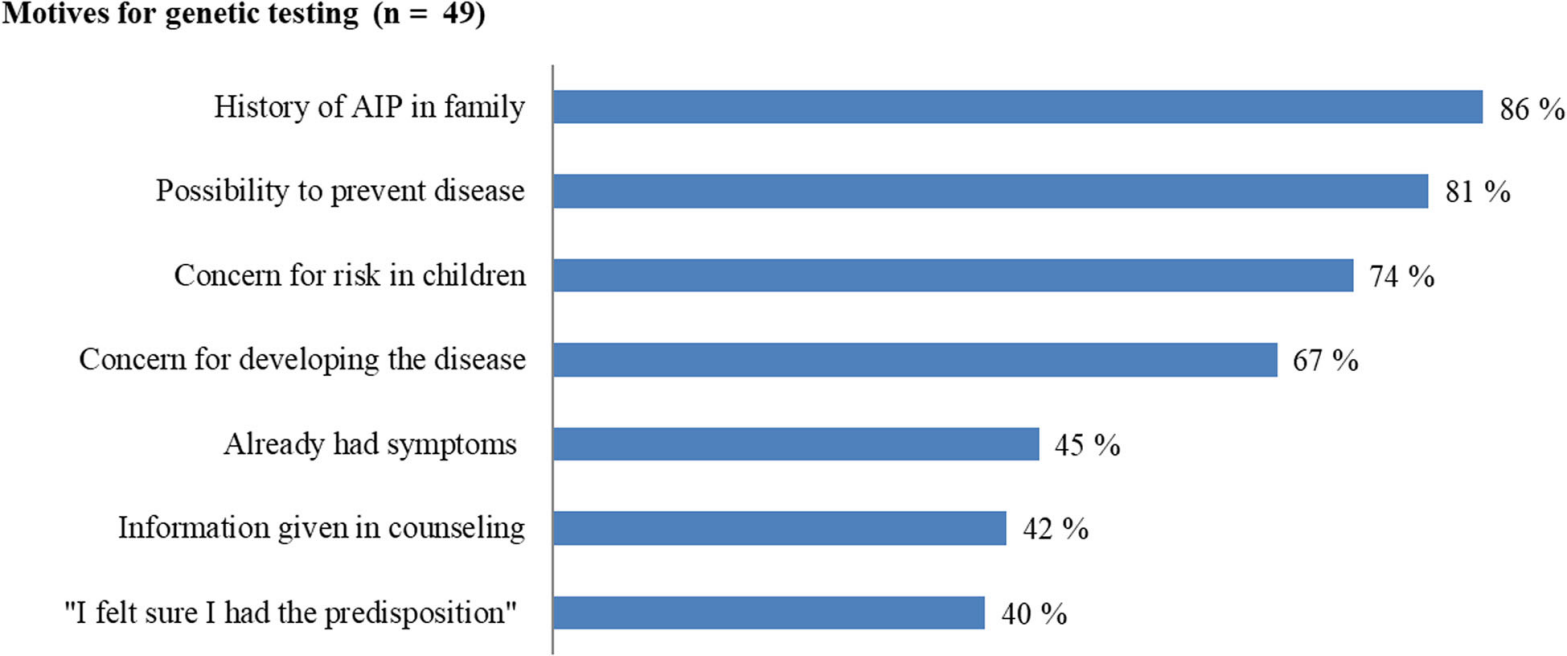
Frequency of respondents who answered “quite important”, “very important”, or “vital” to statements about their motives to undergo genetic testing for AIP. Results are presented as valid percent

Forty-two of the 51 participants who reported receiving genetic counseling had attended only one such session (83%), and for 35 (84%) of these, this had been more than 3 years previously. All groups reported high scores for satisfaction with genetic counseling. Men and women were equally satisfied (Table 3). The most frequent motive for genetic testing (*n* = 49, 2 missing) was having AIP in the family, followed by the ability to prevent activation of AIP and a concern of risk to their children (Fig. 3).

**Table 3:** Satisfaction with genetic counseling with results for the dimensions *Instrumental, Affection* and *Procedural* satisfaction: total scores and scores for men and women separately

Score range 3–12.			
	*n*	Median (IQR^a^)	*p* ^b^
SCS, Instrumental	48	9.5 (7.0, 11.8)	
Women	26	9.0 (6.8, 11.3)	0.23
Men	22	10.0 (9.0, 12.0)	
SCS, Affection	45	11.0 (9.0, 12.0)	
Women	24	10.5 (9.0, 12.0)	0.32
Men	21	12.0 (9.5, 12.0)	
SCS, Procedural	45	11.0 (8.5, 12.0)	
Women	24	10.5 (7.3, 12.0)	0.67
Men	21	11.0 (9.0, 12.0)	

^a^ IQR, interquartile range (i.e., 25th, 75th percentiles)

^b^ Mann–Whitney nonparametric *U* test

## Discussion

In some patients, AIP is a chronic and debilitating disease with severe, recurrent acute attacks and low quality of life [25], while in the majority of patients, acute attacks are sporadic and infrequent. However, in both symptomatic AIP patients and genetically predisposed *HMBS* mutation carriers, life-long adherence to preventive measures is required and the disease carries the risk of severe acute attacks and serious long-term complications.

Self-efficacy is described as self-confidence in coping with various difficulties in life. High self-efficacy is related to higher motivation and a greater effort to solve such difficulties [26]. Having high self-efficacy is likely to be important to individuals with AIP, as lifestyle changes require self-drive and personal initiative. In our study, participants reported a high belief in their own ability to cope and exert control in general. No difference in self-efficacy was found between those self-classified with asymptomatic AIP compared with symptomatic disease. Almost all participants in our study had in some way made changes in their behavior, consistent with the high degree of self-efficacy found among them. Almost all participants reported that they were aware of the risk of using medications listed as porphyrinogenic, which is similar to the results of Andersen et al. [16].

With the exception of avoiding porphyrinogenic medications, recommendations for patients with AIP are in line with general health recommendations, namely sustaining a balanced diet, reducing alcohol and tobacco consumption, and avoiding infections and stress. Therefore, some of the preventive actions reported by the participants might also have been motivated by a desire for a healthier lifestyle in general [16]. Irrespective of the motives, this effort in trying to have a healthier lifestyle might be helpful in preventing acute attacks.

Respondents with self-reported symptomatic AIP reported changes in behavior to a greater extent than the asymptomatic group. It is natural that patients with symptoms they ascribe to their disease would perceive preventive actions as being important [25]. At the same time, experiencing one or several acute attacks could make patients lose their motivation to continue preventive efforts; however, it seems that this is not the case.

That the asymptomatic group reported having made changes in their behavior can be used as an argument for offering predictive testing. By taking preventive measures, the risk of experiencing an acute attack is expected to be reduced. At the same time, it is important to be aware that testing might also lead to some unnecessary worries and pathologising. The list of possible symptoms in AIP is long and can be difficult for the patient to distinguish from more common illnesses [16]. In our study 38/140 (27%) participants reported to experience porphyria-related complaints the present week, which is a very high percentage. Further, 34 participants self-classified as symptomatic reported that they had received genetic counseling (Fig. 1). Patients diagnosed with manifest AIP at NAPOS are offered genetic counseling at the time of diagnosis; however, most genetic counseling sessions are provided in a setting of predictive genetic testing, which in Norway has been mandatory since 2003 [27]. We expect that most of the respondents reporting attending genetic counseling have had predictive genetic testing performed as healthy at-risk relatives. Nevertheless, according to the motives for genetic testing results (Fig. 3), 45% of the study participants reported that symptoms was one of the reasons for testing. It is understandable that patients assume that a possible *HMBS* mutation could explain present symptoms, and our experience is that this often is a challenge in genetic counseling for AIP. Considering the low clinical penetrance for AIP [9, 10], it is unlikely that 34 out of 51 study participants who had attended genetic counseling have symptomatic disease. It is more likely that they have experienced symptoms that they ascribe to their genetic predisposition for AIP. An acute attack is verified by standardized criteria, including the demonstration of increased concentrations of porphobilinogen (PBG) in urine [28]. For instance, when AIP patients are experiencing abdominal pain, which is a common symptom in the general population, AIP is often perceived as the cause, without measurements to demonstrate increased PBG concentrations [14, 29]. It is recommended that symptomatic patients and high-excretors are assessed yearly including measurements of ALA and PBG in urine being performed [14]. It is important to educate patients on the importance of submitting a urine sample for ALA and PBG analysis when they experience symptoms that they consider likely to be related to AIP. It is not beneficial for the patient that symptoms are uncritically attributed to AIP, as it can lead to medicalization, worry, and other serious illnesses might be overlooked.

The least frequently reported change in behavior was avoidance of physical strain. Physical strain is not listed as a triggering factor in several larger studies on acute porphyrias [11, 30] but was reported as a triggering factor, particular in men, in a Northern-Swedish study on 145 manifest AIP patients [12]. It is well known that physical activity has both physical and psychological benefits, including reducing stress levels [31] and psychological stress was reported to be an important attack inducer in an American study of patients with AIP who were experiencing recurrent attacks [25]. Though there are reports that physical strain may be a triggering factor, moderate physical activity is beneficial. To distinguish physical activity from physical strain might however be difficult for the patients, and this should be addressed when counseling this group.

The patients who attended genetic counseling reported satisfaction with all three components of the sessions investigated using the SCS (Table 3). High levels of satisfaction with genetic counseling have also been shown for patients with other diagnoses [32, 33]. For the majority of participants in our study, the counseling sessions took place several years ago. Therefore, the results might not apply to today’s clinical practice, but are still of interest. Satisfied patients are more likely to make use of health services and to carry out medical recommendations [34]. In the decision-making process regarding genetic testing, information provided from the genetic counselor was not experienced as being of great importance. This is perhaps not surprising, as most individuals attending genetic counseling for predictive genetic testing of AIP had made the initiative themselves and it is thus likely that they already had made the decision to undergo testing. Their starting point was usually having a family member with manifest disease, so this is naturally reported as the most important motive (Fig. 3). Respondents also reported that they wanted to clarify their own situation to be able to prevent activation of the disease, which is in line with what they reported to be doing when answering questions about lifestyle changes. It is of interest that concern for developing the disease is reported as a less strong motive for testing than their motivation to be able to prevent the disease. This could be influenced by the fact that 45% reported already having symptoms they ascribed to their likely genetic predisposition, but also with their high self-efficacy, they were focused on the possibilities of avoiding trigger factors to avoid symptomatic disease. The concern for risk in children was also a frequently reported motive.

When patients are diagnosed with AIP at NAPOS, both the physician and the patient receive information about the diagnosis and guidelines for follow-up and treatment, and are informed that NAPOS offers personal counseling, patient courses, identity cards and telephone and e-mail support. It is likely that this information, in addition to the provided counseling, has contributed to study participants’ knowledge on triggering factors and enabled their changes in behavior (Fig. 2). In the self-reported symptomatic group, the respondents who had received genetic counseling reported both a higher level of education and they had more frequently secured employment compared to the group who had not received genetic counseling (Table 1). It is likely that resourceful patients more easily make use of what the health care system has to offer. That attending a specialist porphyria clinic providing advice, management, and counseling can be beneficial in terms of behavioral adjustments has been shown by others [5]. In addition, information about rare diseases is increasingly available, e.g. online. By being informed and experiencing more understanding and competence from health professionals, patients might feel more in control and better looked after.

### Limitations

The design of this study does not allow any conclusions as to whether there is an association between general self-efficacy and the level of preventive actions reported by the participants. The grouping of the respondents into asymptomatic and symptomatic cases of AIP was based on self-reported and self-perceived AIP symptoms. The list of symptoms in AIP is long, and many of the symptoms are the same as those in other more common diseases or general health complaints. It is likely that patients classifying themselves with symptomatic disease attributed other health complaints as being AIP symptoms and in reality should have been classified as “asymptomatic”. Difficulties in separating AIP symptoms from subjective health complaints has been reported by others [16]. Considering the high percentage of patients’ self- classifying as symptomatic in our study, we suspect that if this had been evaluated by standardized criteria including measurements of PBG in urine, fewer patients would have been classified as symptomatic. Also, the study had a retrospective design, which is suitable for studying rare cases with low incidence, but recall bias must always be taken in to consideration [35].

The present study had a response rate of 55%, which is a problem with regard to representability, however, it is in line with average response rates estimated at 53% [36]. The non-responders’ reasons for not answering the questionnaire can only be speculated on, but if they had low self-efficacy and were not motivated for lifestyle changes this would have influenced the conclusions in this study, and can therefore be a limitation to the conclusions drawn. This, however, represents a constant problem in research where participating is based on informed consent.

### Research recommendations

Further investigations into what extent lifestyle contribute to a lower penetrance and expressivity of AIP could yield important knowledge. Continued research is warranted to better understand how counseling can be helpful to this patient group.

## Conclusions

This study indicated high levels of self-efficacy in Norwegian *HMBS* mutation carriers. This positive self-reliance is probably important to apply self-management strategies and might aid in reducing the severity of and/or preventing acute attacks. The respondents reported several preventive measures and behavioral changes after being diagnosed with AIP, the self-reported symptomatic cases to a greater extent than the asymptomatic. The possibility to prevent the disease and to consider the risk for children was important when the respondents decided to be genetically tested. They were highly satisfied with the genetic counseling they had received. Our study indicates that Norwegian *HMBS* mutation carriers have both the knowledge and the self-motivation to make good choices that might aid in preventing activation of the disease. Therefore, providing information, counseling, and education is worthwhile in AIP.

## Additional file

Additional file 1: Questionnaires developed for this study; English translation. Questionnaires developed to elucidate 1. Motives to undergo genetic testing for AIP and 2. Awareness and concern regarding different triggers for AIP (Original language: Norwegian). (DOCX 17 kb)

## Data Availability

The datasets used and/or analyzed during the current study are available from the corresponding author on reasonable request.

## Abbreviations

AIP: Acute intermittent porphyria
ANOVA: One-way analysis of variance
GC: Genetic counseling
GSES: General Perceived Self-efficacy Scale
*HMBS*: Hydroxymethylbilane gene
IQR: Interquartile range
NAPOS: The Norwegian Porphyria Centre
PBG: Porphobilinogen
SCS: Satisfaction with Genetic Counseling Scale
