# Supplementary material for: Self-efficacy and self-management strategies in acute intermittent porphyria
Source: BMC Health Serv Res. 2019 Jul 3;19:444. doi: 10.1186/s12913-019-4285-9 (PMC6607542; doi:10.1186/s12913-019-4285-9)
Supplement: Supplementary file 1 — Questionnaires developed for this study; English translation. Questionnaires developed to elucidate 1. Motives to undergo genetic testing for AIP and 2. Awareness and concern regarding different triggers for AIP (Original language: Norwegian). (DOCX 17 kb) [file 12913_2019_4285_MOESM1_ESM.docx]

**Questionnaires developed for this study; English translation.**

**Part B. Questions regarding genetic counseling**

The next questions (Part B questions 1-22) should only be answered by those having attended genetic counseling and are related to the *counseling session* in connection to genetic testing. If you did not attend such counseling, please continue with part C, page 4

**The next questions are in relation to what motivated you to have a genetic test**

**Please circle the answer that best describes your opinion.**

1 = little importance 2 = somewhat important 3 = quite important

4 = very important 5 = crucial importance 6 = not applicable

1. It was important for me to know if I could ever develop porphyria disease..................... 1 2 3 4 5 6

2. The information I received from the genetic counselor before the test had an impact on my decision........................... 1 2 3 4 5 6

3. I wanted to clarify my own situation to facilitate prevention of the disease.................... 1 2 3 4 5 6

4. I wanted to clarify the situation in regard to my children’s potential risk of disease………1 2 3 4 5 6

5. The presence of porphyria disease in relatives was of significance for my decision............................1 2 3 4 5 6

6. I felt confident I had the genetic disposition for porphyria although I had no symptoms.. 1 2 3 4 5 6

7. I already had symptoms of porpyria when I decided to do genetic testing........................ 1 2 3 4 5 6

**Part C. General questions about porphyria**

The following questions should be answered by everyone.

The following questions are concerning whether you changed your behavior after you received the diagnosis latent or active porphyria

1 = Not at all true   2 = Hardly true 3 = Neither agree, nor disagree

4 = Moderately true 5 = Exactly true 6 = Not applicable

1. I am now more aware of which drugs I am using compared to before.......................1 2 3 4 5 6

2. I am more concerned with eating regular and carbohydrate –rich meals now……… 1 2 3 4 5 6

3. I am more interesting in reducing my alcohol consumption now compared with before…………................1 2 3 4 5 6

4. I try to avoid situations that trigger stress more than I used to.................................. 1 2 3 4 5 6

5. I try to avoid very hard physical exertion more than I used to…………..........................1 2 3 4 5 6

6. I am more aware of avoiding chemical solvents compared to before……….................1 2 3 4 5 6

7. I am more concerned of reducing my tobacco consumption now compared to before ………………………..………..1 2 3 4 5 6
